# Educational intervention on child development in light of the health literacy framework

**DOI:** 10.1590/0034-7167-2024-0439

**Published:** 2026-03-30

**Authors:** Rayara Medeiros Duarte Luz, Nirla Gomes Guedes, Fábia Alexandra Pottes Alves, Francisca Márcia Pereira Linhares, Maria Wanderleya de Lavor Coriolano-Marinus

**Affiliations:** I Universidade Federal de Pernambuco. Recife, Pernambuco, Brazil; II Universidade Federal do Ceará. Fortaleza, Ceará, Brazil

**Keywords:** Child Development, Health Literacy, Health Education, Education, Public Health Professional, Qualitative Research., Desarrollo Infantil, Alfabetización en Salud, Educación en Salud, Educación en Salud Pública Profesional, Investigación Cualitativa.

## Abstract

**Objectives:**

to analyze an educational intervention with primary care healthcare professionals on promoting child development based on the health literacy framework.

**Methods:**

participatory health research, conducted in four stages. The sample consisted of 13 primary care healthcare professionals working in child health surveillance in the municipality of Passira, state of Pernambuco. The intervention took place through workshops. Data analysis was followed according to the stages proposed by Yin.

**Results:**

changes and knowledge were identified. Professionals adopted care actions focused on important aspects of early childhood and improved communication with parents/caregivers through the application of health literacy principles.

**Final Considerations:**

the importance of continuing education in primary care is demonstrated, suggesting the need for further research to strengthen public policies and professional practices within the Brazilian Health System.

## RESUMEN

**Objetivos:**

analizar una intervención educativa con profesionales de la salud de atención primaria para la promoción del desarrollo infantil, basada en el marco de alfabetización en salud.

**Métodos:**

investigación participativa en salud, realizada en cuatro etapas. La muestra estuvo compuesta por 13 profesionales de la salud de atención primaria que trabajan en la vigilancia de la salud infantil en el municipio de Passira, estado de Pernambuco. La intervención se llevó a cabo mediante talleres. El análisis de datos se realizó según las etapas propuestas por Yin.

**Resultados:**

se identificaron cambios y conocimientos. Los profesionales adoptaron medidas de atención centradas en aspectos importantes de la primera infancia y mejoraron la comunicación con los padres/cuidadores mediante la aplicación de principios de alfabetización en salud.

**Consideraciones Finales:**

el estudio demuestra la importancia de la formación continua en Atención Primaria, lo que sugiere la necesidad de realizar más investigaciones para fortalecer las políticas públicas y las prácticas profesionales en el Sistema Único de Salud.

## INTRODUCTION

In Brazil, Primary Health Care (PHC) is the preferred entry point for users into the healthcare service governed by ordinances that direct the comprehensive care of individuals and communities with promotion, prevention, and rehabilitation actions^(1)^. Among the priorities, child health surveillance includes activities aimed at child care, harm prevention, and implementation of interventions to promote qualified health^(2)^.

The child development promotion agenda is emphasized by global initiatives such as the Nurturing Care Framework, which emphasizes the need for integrated actions across health, nutrition, responsive care, learning, safety, and protection. Actions aimed at training healthcare professionals can improve caregivers’ abilities to support their children’s development^(3)^. In addition to Nurturing Care, other global initiatives promote this topic, such as the Sustainable Development Goals for 2030, which align with the creation of strategies to reduce inequities in the world, including reducing mortality and achieving the potential of child development as topics that must be agreed upon intersectorally^(4)^. These aspects can be linked to professional actions in the Family Health Strategy, in which longitudinal monitoring of children and families must be carried out from the perspective of promoting child health in an integrated and comprehensive manner.

In some contexts, caregivers’ needs in PHC have not been met satisfactorily by healthcare professionals. Caregivers do not receive guidance on the availability and use of services for their children’s benefit^(5)^. Therefore, training healthcare professionals working in PHC to guide caregivers in order to achieve longitudinal care in early childhood becomes essential.

Another issue concerns communication between healthcare professionals and children’s primary caregivers. Barriers to communication and access to healthcare services still persist due to inadequate communication between healthcare professionals and users^(6)^.

The health literacy framework considers an individual’s competencies in obtaining health information, understanding, assessing, and applying it to health management. For caregivers to achieve these goals, healthcare professionals must be aligned to address individual needs, thus better enabling them to apply health-related knowledge in their daily lives^(7,8)^.

There are limited studies that portray interventions focused on child development that apply the health literacy framework. Supported interventions can improve the application of knowledge acquired by professionals in their practical actions in the workplace. Furthermore, nursing interventions focused on child development lead to positive outcomes for children’s lives and, consequently, for family relationships^(9,10)^.

## OBJECTIVES

To analyze an educational intervention with Primary Care healthcare professionals on promoting child development based on the health literacy framework.

## METHODS

This is participatory health research, with professionals working in child health surveillance in the PHC context. This type of research addresses problems of interest to a specific population, involving the researcher and the research context in an equal partnership. In this regard, there is proactivity on all sides^(11)^. The study followed four stages: 1. Integrative review preparation^(10)^; 2. Planning; 3. Execution; 4. Educational intervention assessment.

### Study setting

The setting included Family Health Units (FHUs) in the municipality of Passira, located in the countryside of the state of Pernambuco. The municipality is located in the state of Pernambuco, 108 km from the capital, with a territorial area of 326,758 km^2^ and an estimated population of 28,856 inhabitants according to the Brazilian Institute of Geography and Statistics (2021). The population is 100% covered by Family Health teams (FHts), according to data from the *Primeira Infância Primeiro* platform (2021). Of the 14 FHUs, five are located in urban areas and nine in rural areas. One unit in the rural area and one in the urban area were randomly selected for the intervention.

### Study participants

Participants were primary care healthcare professionals who work in child health surveillance within the FHt within the PHC context. The sample selection was intentional^(12)^. The sample consisted of two nurses, four nursing technicians, one dental surgeon, and six Community Health Workers (CHWs), totaling 13 participants. The physicians from both teams did not participate: one due to unavailability and the other due to vacation.

### Educational intervention and data collection

Sociodemographic data were collected to understand participants’ personal (age, gender, sex, race/color, other) and professional characteristics (education, length of experience in primary care, other). Other information regarding child development monitoring was gathered through a semi-structured questionnaire.

After the initial data was organized, the educational intervention began, consisting of five workshops held every 15 days, corresponding to each thematic axis (Chart 1). Planning was based on conceptual, procedural, and attitudinal objectives. At the end of each workshop, healthcare professionals were asked to complete a practical activity to apply the topics covered and provide practical solutions within the healthcare service context. The location of the workshops was pre-determined with participants, but as the meetings progressed, new spaces were used for this purpose. All meetings were recorded using an audio recorder. The mean duration was one to two hours. The workshops were led by the principal investigator (a nurse and master’s student with prior training in the topic and in group leadership), observers (undergraduate nursing students), and group of participants (study participants). Chart 1 presents the workshop topics, teaching resources, practical activities, and guiding questions. The data collected were informed by participants’ narratives, based on previously developed problematizing questions, to enable greater participation, reflection, and sharing of experiences and knowledge.

**Chart 1:** Planning an educational intervention with healthcare professionals on child development and health literacy, Passira, Pernambuco, Brazil, 2023

WORKSHOP TOPIC	STRATEGY/MATERIALS USED	PRACTICAL ACTIVITY	LEADING QUESTIONS
Child development in early childhood	· Audiovisual resources; · Infographics about child development; · Educational video from the United Nations Children’s Fund: “Love, Play and Care - the ABC for the Early Childhood” (*ABC para a Primeira Infância - Amar, Brincar, Cuidar*).	Talking to three parents/caregivers and ask them what their perceptions are about their children’s development.	- What do you understand by child development? - How have I approached child development in my work environment? - How do you guide caregivers on the importance of encouraging their children to develop?
Health literacy in the context of child development surveillance	· Audiovisual resources; · Script for role play dynamics;	Conducting a dialogue with parents/caregivers during child health surveillance and applying the health literacy assumptions.	- What do you understand by health literacy? - How can health literacy be addressed in child development surveillance?
Child health booklet	· Audiovisual resources; · Poster demonstrating the child development monitoring table; · Case study.	Reading the child health booklet and develop an activity with parents/caregivers using the child health booklet. Completing the developmental milestones in the child health booklet during child development monitoring.	- How do you monitor children’s development? - How do you use your child health booklet? - How do I use the child health booklet with parents/caregivers in my workplace?
Communication between professional-parent/caregiver	· Audiovisual resources; · Case study; · “Early childhood is the right time to plant” booklet.	Developing communication strategies with parents/caregivers in their work environment.	- What communication barriers do I identify with parents/caregivers in the workplace? - What strategies do you use to improve communication with parents/caregivers?
Health education and health literacy in child development surveillance	· Audiovisual resources; · Explanation of the integrative review article; · Health literacy poster; · Educational action plan.	Developing an educational action in their work environment focused on child development, considering the health literacy assumptions (access, understand, assess, and apply).	- What do you understand by health education? - How have I developed health education actions that enable the four domains of health literacy?

### Data analysis

The workshop narratives were transcribed into a Microsoft Word^®^ document for later analysis, according to each meeting and location. The analysis was conducted in five interrelated stages: composition, decomposition, recomposition, interpretation, and conclusion^(13)^.

The composition stage involves grouping the data into a specific document containing the statements obtained from each workshop. Decomposition was performed by the first and last authors using Atlas TI software (version 8.0), which assisted in data segmentation and coding. During the recomposition process, new regroupings were developed, and new codes were incorporated. Finally, the results were interpreted, and the final statements were created in dialogue with interpretation. These stages are not linear and should be worked on in a “recursive and iterative” manner^(13)^.

The study was approved by the Research Ethics Committee under Opinion 5,308,561. Participants signed the Informed Consent Form and the Image and Testimony Use Agreement. Their names were protected and identified by professional class (e.g., nurse, nursing technician, etc.), followed by a number indicating the numerical order corresponding to the number of participants.

## RESULTS

Data were synthesized from two FHts, one in an urban area (group A) and the other in a rural area (group B). All participants were female healthcare professionals, aged 21 to 53 years. Their experience in primary care ranged from six months to 27 years.

The data were organized based on the health literacy framework and organized into two categories: 1. Healthcare professionals’ experiences and knowledge about child development in early childhood; and 2. Health literacy assumptions in promoting child development in early childhood.

### Category 1. Healthcare professionals’ experiences and knowledge about child development in early childhood

Professionals discussed how they monitored child development through observations and measurements during scheduled care for children and families in healthcare services. Professionals discussed the concept of child development and its importance in PHC, including measures of growth, motor milestones, and language, recognizing their responsibility for monitoring over time.

*Child development is how they’re developing, whether they’re a person, a healthy child, whether everything is in the right proportions, head, torso. So, there are other things, but, like, what, in the development of* [...] *normal growth, weight. How they’re growing, if they’re growing, if they have proper speech, if they’re talking, if they know how to listen too.* (CHW 2)

*I see development as walking, as talking.* (Nurse 1)

*With a child, everything is standard, they have time to sit, they have time to walk, they have time to talk.* (CHW 3)

The moments in which developmental monitoring occurs were highlighted, particularly the home visit, carried out mainly by CHWs, and childcare consultation, carried out by nurses.

*The child’s development from the moment we go for the postpartum visit, right, from the moment the child is born, it is our obligation to monitor them at the health center* [...] *especially from 0 to 1 year old.* (Nurse 2)

During follow-up, they highlighted childcare as an opportunity not only for anthropometric measurements, but also as a time to identify situations such as delays in developmental milestones, guiding the family through the care process or referring a child to a specialized service.

*Childcare is very important* [...] *many parents think that childcare is about weight; I’m going to take my son to the childcare center, not just about weighing him, just weighing him and that’s it, but they fail to realize that we can see a lot of things.* (Nurse 1)

*Two children with larger head circumferences; could it be genetic? Yes, but what if it’s not genetic? If it’s macrocephaly, if it’s a problem, a tumor, something* [...]. (Nurse 1)

Among the difficulties faced, healthcare professionals mentioned barriers to effective communication with caregivers and the difficulty in accepting and understanding them in situations involving the identification of developmental delays.

*When we talk, they interrupt us right away, they cut us off right away. She kept the door closed to me for a long time, she closed the doors, I knocked* [...] *I called, I called, I insisted, I insisted* [...] *but she never came to the clinic, I never took anyone there, because she doesn’t accept it.* (CHW 1)

*His mother has never agreed to bring him here, and he’s already a young man. We can see he’s not normal like the others. Whenever he watched anything on TV, he’d be like this* [gestures with his hands], *so I went to talk to her, and she almost snapped at me* [referring to his mother]. (CHW 1)

*There are mothers who take their children, and then they don’t like the diagnosis* [...] *it’s probably autism, “I didn’t like the professional, because he said that my son was like that”* [...] *they don’t accept it, they don’t accept it* [...] *this makes things very difficult.* (Nurse 2)

### Category 2. Health literacy assumptions in promoting child development in early childhood

The processes of change in healthcare professionals’ knowledge, skills, and practices based on the health literacy framework encompass the assumptions related to access, understanding, assessment, and application of knowledge and skills related to self-care and health promotion. Professionals’ knowledge, skills, and attitudes regarding child care approaches based on health literacy were identified across all dimensions.

#### Subcategory 1. Access to health information

Participants emphasized that their search for knowledge was based on official websites, such as the Ministry of Health, and social media, such as Instagram^®^. The workshops expanded access to other sources of knowledge about child development, such as increased use of the child health booklet for educational purposes, as well as websites such as the United Nations Children’s Fund (UNICEF) and the *Fundação Maria Cecília Souto Vidigal* (which they learned about through the guides and pamphlets used by the researcher).

*There’s a “blogger”* [social network Instagram^®^]. (CHW 1 - before the intervention)

*A pediatrician I follow on Instagram^®^.* (Nurse 1)

*The ministry itself already has this initiative once a month; each month focuses on a different topic. Now, August is the month of pregnancy, exclusive breastfeeding, so the nurses have already been told that we need to do some activity for pregnant women to guide them towards exclusive breastfeeding.* (Nurse 1 - before the intervention)

*From the internet!* (Nursing technician 2 - before the intervention)

*The child’s appointment says that her son is 6 months old and will be introduced* [...] *I advised her to read the notebook on page 32.* (Nurse 1 - during the educational intervention)

*Only after you gave the papers.* (Nursing technician 3 - during the educational intervention)

#### Subcategory 2. Understanding and assessing information

Participants realized the importance of communicating in plain language as an aspect to be valued in their professional practice, including approaches such as teach-back.

*Literacy, I swore it was breastfeeding* [laughter]. (Nursing technician 3 - during the educational intervention)

*It’s the way we communicate with our patient.* (Nursing technician 4 - during the educational intervention)

*For her to understand, I repeat the question and ask her to do it with me, repeat it and say how she is going to do it so that the child can improve on what she asked me.* (Nursing technician 1 - during the educational intervention)

*It’s about speaking in a simple way so people can understand* [...] *we have to speak the patient’s language.* (CHW 4 - during the educational intervention)

#### Subcategory 3. Assessment of health information

Participants already discussed using tools to address child development issues, using examples from their personal lives or even information from the internet. Based on the new insights gained from the workshops, they recognized the connection with the practical context of the action and the motivation to incorporate more interactive and practical elements into their relationships with mothers.

*Sometimes a piece of information, an example from our lives, something, can add something to someone else’s life, right? Look, my daughter was like that, but it was different, but keep trying.* (Nurse 1 - before the intervention)

*You see that fact on the internet, you think that’s how you should do it, and you do it.* (CHW 5 - before the intervention)

*Since the first meeting that I told you was the first one we participated in that didn’t make us sleepy, that made us want to participate, because the subject covered is the subject that we already use in our daily lives and that we needed to improve, and that was it, it was great.* (CHW 4 - during the educational intervention)

*I believe that we should not only talk, but also work dynamically, so we can understand more. Certainly, the mothers, when they put the little piece of paper there, they understood things better.* (Nursing technician 3 - during the educational intervention)

#### Subcategory 4. Application of health information

Participants were already involved in group activities focused on childcare, such as breastfeeding groups. It is noteworthy that, based on the workshops, the professionals expanded activities aimed at promoting child development within their scope of work in Primary Care, such as guidance on age-appropriate toys, encouragement and positive feedback for mothers, and more comprehensive information during vaccination procedures.

*I always advise mothers if they’re of that age: “Look, start talking, saying little words, right? Put up a drawing that talks about letters”. You have to encourage it, right?* (Nurse 1 - before the intervention)

*Breastfeeding groups start with pregnant women, then mothers, with breastfeeding. We always do this on the day of childcare. In rural areas, we have to use this strategy.* (CHW 4 - before the intervention)

*I congratulated the mother on her effort in feeding the child and asked her to continue offering other fruits.* (Nurse 1 - during the educational intervention)

*In the vaccination room, I provided guidance on the vaccine that would be administered and the possible reactions. I spoke in a simple manner and repeated it several times so that the mother could understand* [...]. (Nursing technician 3 - during the educational intervention)

## DISCUSSION

Based on the objective of analyzing an educational intervention with Primary Care healthcare professionals on child development promotion, based on the health literacy framework, throughout the educational intervention implementation, both the needs and perceptions of healthcare professionals in their actions with caregivers and children were identified, as well as the contribution of new knowledge, skills, and attitudes that could be applied in their daily work.

Actions related to child development were highlighted by the increased involvement of healthcare professionals, such as the use of child health booklets and health literacy principles to improve communication, as well as the pursuit of knowledge in the area of child health. These observed changes resulted in favorable outcomes regarding child health surveillance and communication between healthcare professionals and parents/caregivers.

In the community context, it is important for healthcare professionals to connect collaboratively with the community. Facilitating access for healthcare users through health promotion and prevention actions is reinforced by the Brazilian National Policy for Comprehensive Child Health Care guidelines, which contributes to children’s early development and provides guidance on how and when to encourage it to be expanded in healthcare professionals’ care practices^(14,15)^.

Engaging and addressing child development issues with parents/caregivers establishes shared responsibility for achieving their child’s potential. Extrinsic factors need to be addressed at appropriate times, such as home visits, well-child visits, or other settings, with the goal of guiding child development and health care^(15)^. Health management must plan ongoing education actions that qualify professionals in the Health Care Network^(16)^. However, the professional is responsible for their qualifications when seeking health information that incorporates scientific theory and practice into their care.

The health literacy premises help us understand how professionals access and acquire knowledge for application in their daily work. In the context studied, professionals initially mentioned information acquired through social media such as Instagram^®^, recalling training opportunities on topics relevant to their work. Through the educational process, they were able to retrieve knowledge from reliable sources, such as UNICEF, fostering the use of child health booklets as a tool for mediating written knowledge with caregivers on various topics related to child development.

When obtaining health information, healthcare professionals should assess its coherence and relevance in order to make a judgment regarding the medium and the information accessed. As Sorensen^(17)^ points out, assessment requires critical thinking and judgment of health information. Not only when seeking, but also when providing health information, healthcare professionals must pay attention to how they communicate and understand the domains involved in users’ access, comprehension, assessment, and application of information in healthcare services^(17,18)^.

Reflection on understanding the topic of literacy and child development was present throughout the process, including reports of maternal lack of understanding regarding situations involving delayed skill acquisition by their children. Professionals realized that improved communication could enhance users’ understanding.

Although developmental delays can be identified in healthcare professionals’ practice, there are barriers that hinder child care continuity and referral to specialized services for early treatment. This occurs due to children’s primary caregiver’s lack of acceptance and the need to train professionals on child development and effective communication with caregivers, whether to guide stimuli that promote development or to make referrals when a need is identified that requires special assessment^(19)^.

When applying health information, professionals must be qualified not only in technical skills but also in communication and empathy with healthcare users. Health literacy premises address individual and collective specificities to provide better health conditions and even influence health determinants^(17,18)^.

Healthcare professionals go beyond identifying developmental delays; they must be able to provide guidance appropriate to each child’s age group during early childhood. A study by Yogman *et al*.^(20)^ developed a guide that identifies playful activities that healthcare professionals can advise parents to do with their children to contribute to their development.

In the reality studied, professionals mentioned practical strategies such as guidance on age-appropriate toys, praise and feedback to caregivers, as well as greater attention to the relationship between children and their caregivers.

In professionals’ assessment of the educational intervention, positive aspects stand out regarding the materials provided, methods adopted (active learning methodologies), the availability of group meetings, and the location - because it is close, sometimes in the work environment itself, not requiring travel and, finally, the topic, understanding professionals’ care practice.

A comprehensive approach to early childhood requires healthcare professionals to raise awareness of the integration of numerous aspects that permeate development, such as breastfeeding, vaccination, developmental monitoring through childcare, and support and communication with caregivers. Furthermore, these professionals working in regions far from the capital may have limited access to up-to-date information related to child health care and development, which is appropriate for their work context, based on the needs and challenges faced.

Among the skills and attitudes developed, the highlights included communication supported by written materials, such as the child health booklet, encouragement and feedback activities, practical demonstrations, and support for group activities, which fostered the participation and knowledge of several team members. Another relevant aspect was the immediate perception that the knowledge acquired during the meetings was applied to the real-world work environment.

During the assessment of healthcare professionals’ practices, the adoption of more understandable language was observed, avoiding the use of technical terms that hinder communication with some patients with less health literacy. The teach-back technique^(21)^ was presented in the meetings as a communication strategy in primary care with parents/caregivers. During care practice, professionals reported good adherence to the technique and its ease of use in health guidance.

Continuing education in healthcare services should not be limited to the transfer of information, as professionals need to be proactive in building knowledge and developing their skills and attitudes. More traditional methods commonly used may not be sufficient for healthcare professionals’ real demands^(16)^.

### Study limitations

One limitation is in evaluating the impact of the intervention, since the study did not assess the impacts directed at parents and caregivers, as well as children.

### Contributions to health, nursing or public policy

New theoretical frameworks and approaches can be explored and proposed for new modalities of educational interventions, aimed at acquiring professionals’ learning skills. To this end, as observed in this study, it is important that stakeholders-in this case, healthcare professionals-are included and actively and collaboratively participate in the teaching-learning process, adopting a more interactive and participatory approach that mobilizes them in the educational process. Healthcare professionals must be able to meet the needs of the community they assist and incorporate interventions that address child development perspectives. This study presents an innovative model that can be replicated in the PHC context for healthcare professionals dealing with child care and health promotion.

## FINAL CONSIDERATIONS

Through educational intervention, some changes were obtained in professionals’ knowledge, skills, and attitudes in their care practice to promote adequate development and communication based on health literacy assumptions.

The intervention demonstrated a promising approach for continuing education in healthcare services, especially in Primary Care, by understanding the realities of professionals’ work in this environment and placing them as protagonists of their own learning process in diverse contexts, such as smaller municipalities. The importance of further studies is emphasized, broadening professionals’ perspectives on child development care, addressing other important issues to be addressed within the multiple childhoods and fostering the creation of public policies aimed at strengthening professional practice and services within the Brazilian Health System.

## Data Availability

The research data are available only upon request.
