# Supplementary material for: Educational intervention on child development in light of the health literacy framework
Source: Rev Bras Enferm. 2026 Mar 30;79:e20240439. doi: 10.1590/0034-7167-2024-0439 (PMC13038223; doi:10.1590/0034-7167-2024-0439)
Supplement: Translation

# Translation: Intervenção educativa sobre desenvolvimento infantil à luz do referencial do letramento em saúde

**DOI:** 10.1590/0034-7167-2024-0439pt

**Authors:** Rayara Medeiros Duarte Luz, Nirla Gomes Guedes, Fábia Alexandra Pottes Alves, Francisca Márcia Pereira Linhares, Maria Wanderleya de Lavor Coriolano-Marinus

**Affiliations:** I Universidade Federal de Pernambuco. Recife, Pernambuco, Brasil; II Universidade Federal do Ceará. Fortaleza, Ceará, Brasil

**Keywords:** Desenvolvimento Infantil, Letramento em Saúde, Educação em Saúde, Educação Profissional em Saúde Pública, Pesquisa Qualitativa.

## Abstract

**Objetivos:**

analisar uma intervenção educativa com profissionais de saúde da Atenção Primária sobre promoção do desenvolvimento infantil a partir do referencial do letramento em saúde.

**Métodos:**

pesquisa participativa em saúde, realizada com quatro etapas. A amostra foi composta por 13 profissionais de saúde da Atenção Primária que atuam na vigilância em saúde da criança no município de Passira, estado de Pernambuco. A intervenção ocorreu por *workshops*. Para analisar os dados, seguiram-se as etapas propostas por Yin.

**Resultados:**

foram identificados mudanças e conhecimentos. Os profissionais adotaram ações assistenciais voltadas a aspectos importantes na primeira infância e uma melhor comunicação com pais/mães/cuidadores por intermédio da aplicação dos pressupostos do letramento em saúde.

**Considerações Finais:**

demonstra-se a importância da educação permanente na Atenção Primária, sugerindo-se mais pesquisas para fortalecer políticas públicas e práticas profissionais no Sistema Único de Saúde.

## INTRODUÇÃO

No Brasil, a Atenção Primária à Saúde (APS) é a porta de entrada preferencial dos usuários no serviço de saúde regido por portarias que direcionam o atendimento integral das pessoas e coletividades com ações de promoção, prevenção e reabilitação^(1)^. Das prioridades, a vigilância em saúde da criança compreende as atividades voltadas aos cuidados da criança, prevenção de danos e implementação de intervenções para promoção de uma saúde qualificada^(2)^.

A agenda de promoção do desenvolvimento infantil é enfatizada por iniciativas globais, como a *Framework Nurturing Care*, na qual enfatiza-se a necessidade de ações integradas nos eixos saúde, nutrição, cuidados responsivos, aprendizagem, segurança e proteção. Ações voltadas para capacitar profissionais de saúde podem resultar em melhoria das capacidades dos cuidadores em apoiar o desenvolvimento de seus filhos^(3)^. Além da *Nurturing Care*, outras iniciativas globais fomentam essa temática, a exemplo dos Objetivos de Desenvolvimento Sustentável para 2030, os quais se alinham com a oportunização de estratégias para reduzir iniquidades no mundo, incluindo a redução da mortalidade e o alcance do potencial desenvolvimento infantil como temáticas que devem ser pactuados de maneira intersetorial^(4)^. Esses aspectos podem ser articulados às ações profissionais na Estratégia Saúde da Família, na qual o acompanhamento longitudinal de crianças e famílias deve ser realizado na perspectiva da promoção da saúde da criança de forma integrada e integral.

As demandas dos cuidadores na APS em alguns contextos não têm sido respondidas de forma satisfatória pelos profissionais de saúde. Os cuidadores não recebem orientação sobre a disponibilidade e a utilização dos serviços para benefício do filho^(5)^. Destarte, a capacitação dos profissionais de saúde que estão na APS para orientar cuidadores, de forma a atingir a longitudinalidade do cuidado na primeira infância, torna-se imprescindível.

Outra questão diz respeito à comunicação entre profissional de saúde e cuidador principal da criança. Barreiras no diálogo e acesso dos usuários na rede de assistência à saúde ainda persistem, devido a uma comunicação inadequada entre profissional e usuário de saúde^(6)^.

O referencial do letramento em saúde considera as competências individuais de um indivíduo em obter uma informação sobre saúde, compreensão, avaliação e aplicação para gerenciamento da saúde. Para que um cuidador atinja tais aspectos, os profissionais de saúde devem estar alinhados a atender às especificidades individuais, de forma a melhor capacitá-los para aplicação de conhecimentos relacionados à saúde nas suas vidas diárias^(7,8)^.

Há uma limitação de estudos que retratem intervenções voltadas ao desenvolvimento infantil com aplicação do referencial do letramento em saúde. Intervenções embasadas podem melhorar a aplicação dos conhecimentos obtidos pelos profissionais em suas ações práticas no contexto de trabalho. Além disso, intervenções de enfermagem voltadas ao desenvolvimento infantil implicam desfechos positivos na vida da criança e, consequentemente, na relação familiar^(9,10)^.

## OBJETIVOS

Analisar uma intervenção educativa com profissionais de saúde da Atenção Primária sobre promoção do desenvolvimento infantil a partir do referencial do letramento em saúde.

## MÉTODOS

Pesquisa participativa em saúde, com profissionais que atuam na vigilância em saúde da criança no contexto da APS. Este tipo de pesquisa contempla a resolução de problemas que são de interesse a uma determinada população, envolvendo, em uma parceria equitativa, o pesquisador e o contexto da pesquisa. Nesse sentido, há uma proatividade de todas as partes^(11)^. O estudo seguiu quatro etapas: 1. Elaboração de revisão integrativa^(10)^; 2. Planejamento; 3. Execução; 4. Avaliação da intervenção educativa.

### Local do estudo

O cenário contemplou Unidades de Saúde da Família (USFs) do município de Passira, localizado no interior do estado de Pernambuco. O município está localizado no estado de Pernambuco a 108 km da capital, possuindo uma área territorial de 326,758 km^2^ e uma população estimada de 28.856 habitantes de acordo com o Instituto Brasileiro de Geografia e Estatística (2021). A população é 100% coberta por equipes de Saúde da Família (eSFs) segundo dados da plataforma Primeira Infância Primeiro (2021). Das 14 USFs, cinco estão localizadas na zona urbana, e nove, na zona rural. Selecionou-se, por meio de sorteio aleatório, uma unidade da zona rural e outra da zona urbana para realização da intervenção.

### Participantes do estudo

Os participantes foram os profissionais de saúde da Atenção Primária que compõem a eSF e que atuam na vigilância em saúde da criança no contexto da APS. A seleção da amostra foi realizada de forma intencional^(12)^. A amostra foi composta por duas enfermeiras, quatro técnicas de enfermagem, uma cirurgiã dentista e seis Agentes Comunitários de Saúde (ACS), totalizando 13 participantes. Não participaram os médicos de ambas as equipes: um devido à indisponibilidade para participar e outro por estar de férias.

### Intervenção educativa e coleta de dados

Foram coletados dados sociodemográficos para conhecimento dos participantes quanto às características pessoais (idade, gênero, sexo, raça/cor, outros) e profissionais (formação, tempo de atuação na atenção básica, experiência, outros). Outras informações foram levantadas em relação à vigilância do desenvolvimento infantil por meio de roteiro semiestruturado.

Após os dados iniciais organizados, iniciou-se a intervenção educativa, composta por cinco *workshops*, realizados com uma periodicidade de 15 dias, correspondendo a cada eixo temático (Quadro 1). O planejamento foi baseado em objetivos conceituais, procedimentais e atitudinais. Foi proposto ao final de cada *workshop* que os profissionais de saúde realizassem uma atividade prática para aplicação dos temas trabalhados e resolução prática no contexto dos serviços de saúde. O local dos *workshops* foi pré-estabelecido com os participantes, mas, ao decorrer dos encontros, novos espaços foram utilizados para essa finalidade. Todos os encontros foram gravados por meio de um dispositivo gravador de áudio. A duração média do tempo foi de uma a duas horas. Os *workshops* foram conduzidos pela pesquisadora principal (enfermeira, estudante de mestrado, com capacitação prévia no tema e na condução de grupos), por observadores (estudantes de graduação em enfermagem) e pelo conjunto de pessoas (participantes do estudo). O Quadro 1 apresenta os temas dos *workshops*, recursos didáticos, atividades práticas e perguntas norteadoras. Os dados produzidos para coleta de dados foram subsidiados pelas narrativas dos participantes, a partir de perguntas problematizadoras previamente elaboradas, para possibilitar uma maior participação, reflexão e compartilhamento de vivências e saberes.

**Quadro 1:** Planejamento da intervenção educativa com profissionais de saúde sobre desenvolvimento infantil e letramento em saúde, Passira, Pernambuco, Brasil, 2023

TEMA DO *WORKSHOP*	ESTRATÉGIA/MATERIAIS UTILIZADOS	ATIVIDADE PRÁTICA	PERGUNTAS CONDUTORAS
Desenvolvimento infantil na primeira infância	· Recursos audiovisuais; · Infográficos sobre o desenvolvimento infantil; · Vídeo didático da Fundo das Nações Unidas para a Infância: “Amar, Brincar de Cuidar: ABC para a Primeira Infância”.	Conversar com três pais/mães/cuidadores e pergunte quais suas percepções quanto ao desenvolvimento de seus filhos.	- O que você entende por desenvolvimento infantil? - De que forma tenho abordado o desenvolvimento infantil em meu ambiente de trabalho? - Como você orienta os cuidadores sobre a importância de estimular seus filhos para se desenvolverem?
Letramento em saúde no contexto da vigilância do desenvolvimento infantil	· Recursos audiovisuais; · Roteiro para dinâmica *role play*;	Realizar um diálogo com pais/mães/cuidadores durante a vigilância de saúde da criança e aplique os pressupostos do letramento em saúde.	- O que você entende por letramento em saúde? - Como o letramento em saúde pode ser abordado na vigilância do desenvolvimento infantil?
Caderneta de saúde da criança	· Recursos audiovisuais; · Cartaz demonstrativo da tabela de acompanhamento do desenvolvimento infantil; · Estudo de caso.	Ler a caderneta de saúde da criança, e desenvolver uma atividade com pais/mães/cuidadores utilizando a caderneta de saúde da criança. Preencher os marcos do desenvolvimento na caderneta de saúde da criança durante acompanhamento/monitoramento do desenvolvimento infantil.	- Como você acompanha o desenvolvimento das crianças? - De que forma você utiliza a caderneta de saúde da criança? - Como utilizar a caderneta de saúde da criança com pais/mães/cuidadores em meu ambiente de trabalho?
Comunicação entre profissional-pai/mãe/cuidador	· Recursos audiovisuais; · Estudo de caso; · Cartilha “A primeira infância é o tempo certo de plantar”.	Desenvolver estratégias de comunicação com pais/mães/cuidadores em seu ambiente de trabalho.	- Quais barreiras de comunicação identifico com pais/mães/cuidadores no ambiente de trabalho? - Quais estratégias você utiliza para melhorar a comunicação com pais/mães/cuidadores?
Educação em saúde e letramento em saúde na vigilância do desenvolvimento infantil	· Recursos audiovisuais; · Explanação do artigo de revisão integrativa; · Cartaz do letramento em saúde; · Plano de ação educativa.	Desenvolver uma ação educativa em seu ambiente de trabalho voltada para o desenvolvimento infantil, considerando os pressupostos do letramento em saúde (acessar, compreender, avaliar e aplicar).	- O que você entende por educação em saúde? - De que forma tenho desenvolvido ações de educação em saúde que viabilizem os quatro domínios do letramento em saúde?

### Análise de dados

As narrativas dos *workshops* foram transcritas em documento *Microsoft Word^®^* de acordo com cada encontro e local de realização para posterior análise. A análise foi realizada em cinco etapas interrelacionadas: composição, decomposição, recomposição, interpretação e conclusão^(13)^.

A etapa de composição denota o agrupamento dos dados em documento específico com as referidas falas obtidas de acordo com cada *workshop*. A decomposição foi realizada pela primeira autora e última autora, com auxílio do *software* Atlas TI (versão 8.0), o qual auxiliou na segmentação dos dados e codificação. Na recomposição, novas reagrupações foram elaboradas, e novos códigos foram sendo incorporados. Por fim, seguiram-se a interpretação dos resultados e a conclusão na criação de enunciados em diálogo com a interpretação. Essas etapas não ocorrem de forma linear, devendo ser trabalhadas de forma “recursiva e iterativa”^(13)^.

O estudo foi aprovado pelo Comitê de Ética em Pesquisa, sob Parecer n^o^ 5.308.561. As participantes assinaram o Termo de Consentimento Livre e Esclarecido e o Termo de Uso de Imagem e Depoimento. Os nomes foram preservados e identificados por meio da classe profissional (ex., enfermeiro, técnico de enfermagem, *etc*.), seguido de uma numeração, que indica a ordem numeral que corresponde ao quantitativo de participantes.

## RESULTADOS

Os dados foram sintetizados a partir das duas eSFs, sendo uma na zona urbana (grupo A) e outra zona rural (grupo B). Todos os participantes eram profissionais de saúde, do sexo feminino, com idade de 21 a 53 anos de idade. A experiência na atenção básica variou de seis meses a 27 anos.

Os dados foram organizados a partir do referencial do letramento em saúde e organizados em duas categorias: 1. Vivências e saberes dos profissionais de saúde sobre desenvolvimento infantil na primeira infância; e 2. Pressupostos do letramento em saúde na promoção do desenvolvimento infantil na primeira infância.

### Categoria 1. Vivências e saberes dos profissionais de saúde sobre desenvolvimento infantil na primeira infância

Os profissionais abordaram como realizavam o monitoramento do desenvolvimento infantil por meio de observações e medidas durante o cuidado programado das crianças e famílias no serviço de saúde. A compreensão do conceito de desenvolvimento infantil e sua importância de ser retratada na APS foram abordadas pelos profissionais como medidas de crescimento, marcos motores e linguagem, reconhecendo como sua responsabilidade o acompanhamento ao longo do tempo.

*Desenvolvimento infantil é como é que tá se desenvolvendo ele, se tá uma pessoa, uma criança sadia, se tá tudo na medida certa, de cabeça, tronco. Assim, tem outras coisas, mas, assim, o que, no desenvolvimento da* [...] *do crescimento normal, peso. Como é que ele tá crescendo, se tá, se ele tem a fala direitinho, se tá falando, se tá, sabe escutar também.* (ACS 2)

*Eu vejo o desenvolvimento sendo andar, sendo falar.* (Enfermeira 1)

*Criança tudo é padrão, ela tem o tempo de sentar, ela tem o tempo de andar, ela tem o tempo de falar.* (ACS 3)

Os momentos nos quais ocorre a vigilância do desenvolvimento foram destacados, particularmente a visita domiciliar, realizada principalmente pelos ACS, e a consulta de puericultura, realizada pelos enfermeiros.

*O desenvolvimento infantil da criança desde quando a gente foi fazer a visita puerperal, né, desde aquele momento que a criança nasce, ela é nossa obrigação de acompanhamento no posto de saúde* [...] *principalmente de 0 a 1 ano.* (Enfermeira 2)

Durante o acompanhamento, destacaram a puericultura como oportunidade não apenas para as medidas antropométricas, mas como momento para identificação de situações como atrasos nos marcos do desenvolvimento, conduzindo a família no processo de cuidar ou encaminhando para um serviço especializado.

*A pueri é muito importante* [...] *muitos pais acham que a pueri é o peso; vou levar meu menino pra puericultura não que é só pesar, só pesou e pronto, mas elas não conseguem perceber que a gente consegue ver muita coisa.* (Enfermeira 1)

*Duas crianças que estão com o perímetro cefálico maior; pode ser genético? Pode, mas e se não for genético? Se for uma macrocefalia, se for um problema, um tumor, alguma coisa* [...]. (Enfermeira 1)

Entre as dificuldades enfrentadas, os profissionais de saúde mencionaram as barreiras para a comunicação efetiva com os cuidadores e a dificuldade para aceitação e compreensão destes diante de situações que envolviam a identificação de atrasos no desenvolvimento.

*Quando a gente fala, elas interrompem logo a gente, elas cortam logo. Ela ficou de porta fechada um bom tempo pra mim, fechava as portas, eu batia* [...] *chamava, chamava, insistia, insistia* [...] *mas nunca veio pra posto, nunca levei ninguém lá, porque não aceita.* (ACS 1)

*A mãe até hoje nunca aceitou trazer ele, e já tá um rapazinho. A gente percebe que ele não é normal feito os outros. Quando ele via qualquer coisa na frente da televisão, ele ficava assim* [faz gesticulações com as mãos]*, aí eu fui conversar com ela, ela só faltou me engolir* [referindo-se à mãe]. (ACS 1)

*Tem mãe que leva a criança, aí não gosta do diagnóstico* [...] *provavelmente é autismo, “não gostei do profissional, porque disse que meu filho era isso”* [...] *não aceita, não aceita* [...] *isso dificulta muito.* (Enfermeira 2)

### Categoria 2. Pressupostos do letramento em saúde na promoção do desenvolvimento infantil na primeira infância

Os processos de mudanças nos conhecimentos, habilidades e práticas dos profissionais de saúde a partir do referencial do letramento em saúde abarcam os pressupostos relacionados ao acesso, compreensão, avaliação, e aplicação de conhecimentos e habilidades relacionados ao autocuidado e promoção da saúde. Os conhecimentos, habilidades e atitudes dos profissionais para abordagem de cuidados com a criança embasados no letramento em saúde foram identificados em todas as dimensões.

#### Subcategoria 1. Acesso às informações de saúde

As participantes destacaram que a busca por conhecimentos era embasada em sites oficiais, como o do Ministério da Saúde, e redes sociais, como o *Instagram^®^*. A partir dos *workshops*, ampliaram-se outros meios de acesso aos conhecimentos sobre o desenvolvimento infantil, como uma maior utilização da caderneta de saúde da criança, com propósito educativo, além de *sites*, como o da Fundo das Nações Unidas para a Infância (UNICEF) e da Fundação Maria Cecília Souto Vidigal (os quais tomaram conhecimento por meio dos guias e panfletos utilizados pela pesquisadora).

*Tem uma “blogueirazinha”* [rede social *Instagram^®^*]. (ACS 1 - antes da intervenção)

*Uma médica pediatra que eu acompanho no Instagram^®^*. (Enfermeira 1)

*O próprio ministério, ele já tem essa iniciativa uma vez ao mês; cada mês é voltado pra uma parte. Pronto, agora agosto é gestante, aleitamento exclusivo, então já foi passado pras enfermeiras que a gente precisa fazer alguma atividade pras gestantes pra orientar a amamentação exclusiva.* (Enfermeira 1 - antes da intervenção)

*Da internet!* (Técnica de enfermagem 2 - antes da intervenção)

*A consulta de pueri e diz que seu filhinho tá com 6 meses, já vai introduzir* [...] *eu orientei ela ler a caderneta na página 32.* (Enfermeira 1 - durante a intervenção educativa)

*Só depois que você deu os papéis.* (Técnica de enfermagem 3 - durante a intervenção educativa)

#### Subcategoria 2. Compreensão e avaliação das informações

As participantes perceberam a importância da comunicação em linguagem simples como um aspecto a ser valorizado na sua prática profissional, incluindo abordagens como o *teach-back*.

*Letramento, eu jurava que era aleitamento materno* [risos]. (Técnica de enfermagem 3 - durante a intervenção educativa)

*É a maneira como a gente se comunica com o nosso paciente.* (Técnica de enfermagem 4 - durante a intervenção educativa)

*Pra ela compreender, eu repito a pergunta e peço pra que ela faça comigo, repita e diga como é que ela vai fazer pra que a criança tenha melhoria no que ela me perguntou.* (Técnica de enfermagem 1 - durante a intervenção educativa)

*É falar de forma simples pro pessoal entender* [...] *a gente tem que falar a linguagem do paciente.* (ACS 4 - durante a intervenção educativa)

#### Subcategoria 3. Avaliação das informações em saúde

As participantes abordaram já utilizar elementos voltados à abordagem das temáticas ligadas ao desenvolvimento infantil, a partir de exemplos da vida pessoal ou ainda pelo uso de informações da internet. A partir dos novos conhecimentos dos *workshops*, as perceberam a relação com o contexto prático da ação e a motivação para agregar elementos mais interativos e práticos na relação com as mães.

*Às vezes uma informação, um exemplo da nossa vida, alguma coisa, pode acrescentar na vida do outro, né? Olha, a minha menina era assim, mas foi diferente, mas vá tentando.* (Enfermeira 1 - antes da intervenção)

*Vê lá na internet aquele fato, acha que aquilo ali é pra fazer daquele jeito e faz.* (ACS 5 - antes da intervenção)

*Desde o primeiro encontro que eu disse a você foi a primeira que a gente participou que não deu sono, que deu vontade da gente participar, porque o assunto abordado é o assunto que a gente já usa no dia a dia e que a gente precisava se aprimorar, e foi isso, foi ótimo*. (ACS 4 - durante a intervenção educativa)

*Eu avalio que não só a gente deve falar, mas trabalhar de forma dinâmica, né, que a gente consegue compreender mais. Com certeza, as mães, na hora que colocaram ali o papelzinho, elas compreenderam melhor as coisas.* (Técnica de enfermagem 3 - durante a intervenção educativa)

#### Subcategoria 4. Aplicação das informações em saúde

As participantes já realizavam ações em grupo voltadas ao cuidado com as crianças, como o grupo de aleitamento materno. Destaca-se que, a partir dos *workshops*, as profissionais ampliaram atividades voltadas à promoção do desenvolvimento infantil dentro do seu escopo de atuação na Atenção Primária, como as orientações acerca de brinquedos apropriados à idade da criança, encorajamento e *feedback* positivo para as mães, e informações mais completas durante a realização do procedimento de vacinação.

*Sempre oriento as mães se for da idade: “Olhe, comece a falar, dizer palavrinhas, né? Coloque desenho que fale das letrinhas”. Tem que estimular, né?* (Enfermeira 1 - antes da intervenção)

*Grupo de aleitamento materno, a gente já começa com a gestante, aí depois as mães, com aleitamento materno. A gente faz sempre no dia da pueri. Na zona rural, a gente tem que usar dessa estratégia.* (ACS 4 - antes da intervenção)

*Eu parabenizei a mãe pelo esforço de dar comida a criança e pedi que ela continuasse oferecendo outras frutas.* (Enfermeira 1 - durante a intervenção educativa)

*Na sala de vacina, orientei sobre a vacina que seriam aplicadas e as possíveis reações, falei de maneira simples, repeti várias vezes para que a mãe pudesse entender* [...]. (Técnica de enfermagem 3 - durante a intervenção educativa)

## DISCUSSÃO

A partir do objetivo de analisar uma intervenção educativa com profissionais de saúde da Atenção Primária sobre promoção do desenvolvimento infantil, a partir do referencial do letramento em saúde, foram identificados, ao longo da implementação da intervenção educativa, tanto as necessidades e percepções dos profissionais de saúde nas suas ações com os cuidadores e crianças quanto a contribuição dos novos conhecimentos, habilidades e atitudes que puderam ser aplicados no seu cotidiano de trabalho.

As ações relacionadas ao desenvolvimento infantil foram destacadas a partir do maior envolvimento dos profissionais de saúde, como o uso da caderneta de saúde da criança e dos pressupostos do letramento em saúde para aprimorar a forma de comunicação, assim como a busca de conhecimentos na área da saúde da criança. Essas mudanças observadas resultaram em desfechos favoráveis no que tange à vigilância em saúde da criança e à comunicação entre profissional de saúde e pai/mãe/cuidador.

No contexto comunitário, importa que o profissional de saúde se vincule com a comunidade de maneira colaborativa. A facilitação do acesso dos usuários de saúde por meio de ações de promoção da saúde e prevenção é reforçada pelas diretrizes da Política Nacional de Atenção Integral à Saúde da Criança, no sentido de contribuir para o desenvolvimento infantil das crianças, orientando sobre como e quando estimular deve ser ampliada na prática assistencial dos profissionais de saúde^(14,15)^.

O envolvimento e a abordagem de temas relacionados ao desenvolvimento infantil com pais/mães/cuidadores estabelecem uma corresponsabilização no alcance do potencial desenvolvimento do filho. Os fatores extrínsecos precisam ser trabalhados em momentos oportunos, como a visita domiciliar, consultas de puericultura ou outros espaços, com o objetivo de conduzir o desenvolvimento da criança e cuidados em saúde^(15)^. A gestão em saúde deve planejar ações de educação permanente que qualifiquem os profissionais da Rede de Atenção à Saúde^(16)^. Não obstante, o profissional é responsável pela sua qualificação ao buscar informações em saúde que incorporem teoria e prática científica na sua assistência.

Os pressupostos da alfabetização em saúde auxiliam na compreensão das formas pelas quais os profissionais acessam e obtêm conhecimento para aplicação no seu cotidiano de trabalho. Na realidade estudada, os profissionais mencionaram, inicialmente, as informações adquiridas em mídias sociais como o *Instagram^®^*, relembrando oportunidades de capacitação sobre temas que permeiam a sua atuação. A partir do processo educativo realizado, puderam resgatar formas de conhecimento provenientes de fontes confiáveis, como a UNICEF, despertando para o uso da caderneta de saúde da criança como instrumento de mediação do conhecimento escrito com os cuidadores das crianças em diferentes temáticas que permeiam o desenvolvimento infantil.

O profissional de saúde deve, ao obter uma informação de saúde, buscar avaliar a coerência e pertinência da informação, de modo a realizar um julgamento quanto ao meio e à informação acessada. Conforme apresentado por Sorensen^(17)^, avaliar requer criticidade e julgamento das informações em saúde. Não apenas para buscar, mas, ao disponibilizar uma informação em saúde, o profissional de saúde precisa se atentar à forma de se comunicar e compreender os domínios que envolvam o acesso, compreensão, avaliação e aplicação da informação pelo usuário no serviço de saúde^(17,18)^.

A reflexão sobre a compreensão do tema letramento e desenvolvimento infantil esteve presente ao longo de todo o processo, inclusive com os relatos da falta da compreensão materna sobre situações que envolviam o atraso na aquisição de habilidades por parte dos filhos. Os profissionais puderam perceber que a melhoria na comunicação poderia aprimorar a compreensão dos usuários.

Embora atrasos no desenvolvimento possam ser identificados na prática dos profissionais de saúde, há entraves que dificultam a continuidade do cuidado da criança e o encaminhamento a serviços especializados para tratamento precoce. Isso ocorre devido à não aceitação do cuidador principal da criança e à necessidade de capacitar o profissional sobre o desenvolvimento infantil e a comunicação efetiva com o cuidador, seja para orientar estímulos que favoreçam o desenvolvimento ou para o encaminhamento, quando identificada alguma demanda que precise de avaliação especial^(19)^.

Ao aplicar uma informação em saúde, o profissional precisa estar habilitado além das competências técnicas, mas de comunicação e acolhimento com o usuário de saúde. Os pressupostos do letramento em saúde atendem às especificidades individuais e coletivas para prover melhor condições de saúde e até mesmo interferir nos determinantes em saúde^(17,18)^.

O profissional de saúde vai além da identificação de atrasos no desenvolvimento, pois importa que esteja apto a orientar estímulos de acordo com cada faixa etária da criança durante a primeira infância. Estudo desenvolvido por Yogman *et al*.^(20)^ desenvolveu um guia que identifica atividades lúdicas que os profissionais de saúde podem aconselhar os pais a fazerem com os seus filhos para contribuir para o seu desenvolvimento.

Na realidade estudada, os profissionais mencionaram estratégias práticas como orientações de brinquedos apropriados para a idade, elogios e *feedback* aos cuidadores, além de uma maior atenção para relação entre as crianças e seus cuidadores.

Na avaliação dos profissionais sobre a intervenção educativa, ressaltam-se os aspectos positivos quanto aos materiais disponibilizados, métodos adotados (metodologias ativas de aprendizagem), à disposição dos encontros em grupos, e local - por ser próximo, por vezes no próprio ambiente de trabalho, não necessitando de locomoção e, por fim, pela temática, compreendendo a prática assistencial dos profissionais.

O olhar sobre a primeira infância em sua totalidade requer dos profissionais de saúde a sensibilização para integração de inúmeros aspectos que permeiam o desenvolvimento, como aleitamento materno, vacinação, vigilância do desenvolvimento por meio da puericultura, e apoio e comunicação com os cuidadores. Além disso, o acesso dessas profissionais que trabalham em regiões distantes da capital pode ficar comprometido quanto à atualização das informações relacionadas à atenção à saúde da criança e desenvolvimento infantil, sendo oportuna na sua realidade de trabalho a partir das necessidades e desafios enfrentados.

Quanto às habilidades e atitudes desenvolvidas, destacam-se a comunicação com apoio de materiais escritos, como a caderneta de saúde da criança, atividades de encorajamento e *feedback*, demonstrações práticas e de apoio para atividades em grupo, as quais favorecem a participação e conhecimento de vários profissionais da equipe. Outro ponto relevante foi a percepção imediata de aplicação dos conhecimentos construídos ao longo dos encontros para a realidade de trabalho.

Durante a avaliação da prática dos profissionais de saúde observa-se a adoção de uma linguagem mais compreensível, sem uso de termos técnicos que dificultam a relação com alguns usuários que apresentam um menor letramento em saúde. A técnica *teach-back* ^(21)^ foi apresentada nos encontros como estratégia de comunicação na atenção básica com pais/mães/cuidadores. Durante prática assistencial, referida pelos profissionais, nota-se uma boa adesão da técnica e facilidade em sua utilização nas orientações de saúde.

A educação permanente nos serviços de saúde não deve se limitar a repassar informações, pois os profissionais precisam ser proativos na construção do conhecimento e desenvolvimento de suas habilidades e atitudes. Os métodos mais tradicionais comumente utilizados podem não ser suficientes para as reais demandas dos profissionais nos serviços de saúde^(16)^.

### Limitações do estudo

Uma limitação está na avaliação do impacto da intervenção, uma vez que o estudo não avaliou os impactos direcionados aos pais e cuidadores, bem como às crianças.

### Contribuições para a área da saúde, enfermagem ou políticas públicas

As novas formas e referenciais teóricos podem ser experienciados e propostos para novas modalidades de intervenções educativas, voltadas à aquisição de competências de aprendizagem dos profissionais. Para isso, conforme observado no presente estudo, é importante que os atores de interesse, nesse caso, os profissionais de saúde, estejam inclusos e participem de forma ativa e colaborativa do processo de ensino-aprendizagem, uma modalidade mais interativa e participativa que os mobilize no processo de educação. O profissional de saúde deve estar apto a atender às necessidades da comunidade atendida e incorporar intervenções que alcancem as perspectivas do desenvolvimento infantil. Este estudo apresenta inovação quanto ao modelo empregado, podendo ser replicado no contexto da APS para profissionais de saúde que lidam com o cuidado e promoção da saúde da criança.

## CONSIDERAÇÕES FINAIS

Por meio da intervenção educativa, algumas mudanças foram obtidas nos conhecimentos, habilidades e atitudes dos profissionais em sua prática assistencial para promoção do desenvolvimento adequado e uma comunicação pautada nos pressupostos do letramento em saúde.

A intervenção mostrou uma proposta promissora para educação permanente nos serviços de saúde, em especial na Atenção Primária, ao compreender a realidade de atuação dos profissionais nesse ambiente, trazendo-os como protagonistas do seu processo de aprendizagem em contextos diversificados, como municípios de menor porte populacional. Ressalta-se a importância de novos estudos que ampliem a perspectiva dos profissionais acerca dos cuidados voltados ao desenvolvimento infantil, abordando outras questões importantes a serem solucionadas dentro das múltiplas infâncias e que favoreçam a criação de políticas públicas direcionadas ao fortalecimento da prática profissional e dos serviços do Sistema Único de Saúde.

## Data Availability

Os dados de pesquisa só estão disponíveis mediante solicitação.
